# Elevated serum IL-10/IL-6 ratio as a novel biomarker for secondary central nervous system lymphoma and poor prognosis in DLBCL

**DOI:** 10.3389/fimmu.2025.1656044

**Published:** 2025-08-13

**Authors:** Jingjing Wen, Xingyu Nie, Qiaolin Zhou, Yiping Liu, Jing Yue, Ya Zhang, Jing Su, Xiaogong Liang, Fang Xu

**Affiliations:** Department of Hematology, Mianyang Central Hospital, School of Medicine, University of Electronic Science and Technology of China, National Health Commission Key Laboratory of Nuclear Technology Medical Transformation, Mianyang, Sichuan, China

**Keywords:** diffuse large B-cell lymphoma, central nervous system, interleukin-10, interleukin-6, prognosis

## Abstract

**Introduction:**

Diffuse large B-cell lymphoma (DLBCL) is the most common subtype of non-Hodgkin lymphoma and carries a poor prognosis when it involves the central nervous system (CNS), a condition known as secondary CNS lymphoma (SCNSL). Although the CNS International Prognostic Index (CNS-IPI) is used to estimate SCNSL risk, its limited sensitivity highlights the need for more reliable biomarkers to improve risk stratification and enable earlier intervention.

**Methods:**

We evaluated pretreatment levels of interleukin-10 (IL-10) and interleukin-6 (IL-6) in both peripheral blood (PB) and cerebrospinal fluid (CSF), and compared clinical characteristics between DLBCL patients with and without SCNSL.

**Results:**

Fifty-six newly diagnosed DLBCL patients who received at least two treatment cycles were included. Compared to patients without CNS relapse, those with SCNSL exhibited distinct clinical features: more frequent B symptoms, increased bone marrow involvement, and lower B-cell lymphoma 6 (BCL6) immunohistochemical positivity. Biochemically, SCNSL patients showed elevated serum IL-10 levels, higher serum IL-10/IL-6 ratios, increased CSF IL-10 concentrations, and markedly elevated CSF IL-10/IL-6 ratios. On multivariate analysis, a serum IL-10/IL-6 ratio ≥2.30 independently predicted both progression-free survival (PFS) (HR = 7.300, *p* = 0.010) and SCNSL development (OR = 43.200, *p* = 0.001). Notably, time to CNS relapse did not significantly differ between high- and non-high-risk groups defined by CNS-IPI (χ² = 1.654, *p* = 0.198). However, incorporating the serum IL-10/IL-6 ratio into the CNS-IPI yielded a refined scoring model—CNS-IPI-ratio—where the high-risk group had a significantly shorter median time to CNS relapse compared to the non-high-risk group (22.32 vs. 40.35 months; χ² = 5.680, *p* = 0.017). Although survival outcomes were similar between high- and non-high-risk groups based on the NCCN-IPI alone (*p* > 0.05), adding the serum IL-10/IL-6 ratio identified significantly poorer outcomes in high-risk patients (PFS: *p* = 0.046; OS: *p* = 0.023). Furthermore, SCNSL patients demonstrated significantly higher CSF IL-10/IL-6 ratios compared to non-SCNSL patients and controls (67.88 vs. 0.74–0.79, *p* < 0.05), with this ratio strongly correlating with CSF lactate dehydrogenase (LDH) levels (r = 0.625, *p* = 0.006).

**Conclusion:**

In DLBCL, an elevated serum IL-10/IL-6 ratio at diagnosis independently predicts disease progression and SCNSL risk. Incorporating this biomarker enhances the prognostic utility of both CNS-IPI and NCCN-IPI models. Additionally, the markedly elevated CSF IL-10/IL-6 ratio in SCNSL patients suggests potential diagnostic value for CNS involvement, warranting further investigation.

## 1 Introduction

Diffuse large B-cell lymphoma (DLBCL) is the most common aggressive subtype of non-Hodgkin lymphoma (NHL) in adults, accounting for 30–40% of cases worldwide (1). Although frontline regimens such as R-CHOP have improved clinical outcomes, approximately 2–5% of patients still develop secondary central nervous system lymphoma (SCNSL) (2), with rates increasing to 10–12% in high-risk subgroups (3). SCNSL is a rare but often fatal complication, typically occurring within 7–8 months of initial diagnosis (4–6). Patients with central nervous system (CNS) involvement exhibit dismal prognoses, with a median overall survival (OS) of only 3.4 months (7). Given the poor outcomes associated with CNS relapse, early identification of high-risk DLBCL patients is crucial for guiding preventive CNS-directed interventions and improving survival.

In 2016, Schmitz et al. (8) developed the Central Nervous System International Prognostic Index (CNS-IPI) as a tool for stratifying CNS relapse risk in DLBCL patients. The CNS-IPI has since been incorporated into National Comprehensive Cancer Network (NCCN) guidelines to assess the likelihood of CNS involvement (9). The reported 2-year CNS relapse rates were 0.6%, 3.4%, and 10.2% for patients in low-, intermediate-, and high-risk groups, respectively (8). However, follow-up data revealed that 55% of patients who developed CNS relapse belonged to the high-risk group, 40% to the intermediate-risk group, and 5% to the low-risk group (8). Moreover, real-world data over a 3-year follow-up period reported a higher-than-expected CNS relapse rate of 10.1% in the intermediate-risk category (5). These findings underscore the limited sensitivity of CNS-IPI in accurately identifying DLBCL patients at elevated risk of SCNSL. To improve predictive accuracy, incorporation of biologically relevant biomarkers has been proposed.

Recent studies have highlighted the role of cytokines within the tumor microenvironment. Interleukin-10 (IL-10), an anti-inflammatory cytokine produced by monocytes, macrophages, and tumor cells, contributes to the regulation of the blood–brain barrier and promotes lymphoma progression, thereby facilitating CNS infiltration (1, 10). Interleukin-6 (IL-6), a key growth factor for B cells, supports tumor growth and metastasis through its secretion by DLBCL cells and the surrounding stroma (11). While the diagnostic value of cerebrospinal fluid (CSF) IL-10 and IL-10/IL-6 ratios has been established in primary CNS lymphoma (PCNSL) (12–15), their utility in predicting CNS relapse in SCNSL remains underexplored. Unlike PCNSL, which presents with isolated CNS involvement, SCNSL requires early risk stratification before CNS dissemination occurs, highlighting the need for minimally invasive predictive biomarkers. Prior studies have shown significantly elevated serum IL-6 and IL-10 levels in DLBCL patients compared to healthy individuals (10, 16), with elevated cytokine levels correlating with shorter progression-free survival (PFS) and OS (10). However, the prognostic and predictive value of serum IL-6 and IL-10—alone or in combination with CNS-IPI—remains insufficiently characterized in the context of SCNSL.

In this study, we analyzed serum and CSF levels of IL-10 and IL-6 at diagnosis to assess their clinical significance in predicting disease progression and CNS relapse in DLBCL. We also examined correlations between serum and CSF cytokine levels and evaluated whether incorporating these biomarkers into the CNS-IPI model could enhance its predictive performance for SCNSL.

## 2 Patients and methods

### 2.1 Patients

We prospectively enrolled 56 newly diagnosed DLBCL patients aged ≥18 years, who were diagnosed and treated at the Department of Hematology, Mianyang Central Hospital between January 2020 and January 2024. Diagnosis and treatment adhered to the 2016 WHO Classification of Tumors of Haematopoietic and Lymphoid Tissues (17) and the NCCN Clinical Practice Guidelines in Oncology: B-Cell Lymphomas (9). All patients received at least three cycles of chemotherapy. Exclusion criteria included: (a) primary CNS lymphoma (PCNSL); (b) active non-lymphoma malignancies; (c) secondary CNS lymphoma (SCNSL) at diagnosis; or (d) pregnancy or lactation. SCNSL was defined as CNS involvement secondary to systemic lymphoma (18). CNS involvement was diagnosed through imaging, detection of lymphoma cells in the CSF, and/or characteristic clinical symptoms (18, 19). All patients underwent standardized imaging evaluations, including whole-body fluorodeoxyglucose positron emission tomography-computed tomography (PET-CT) or contrast-enhanced CT of the chest, abdomen, and pelvis. Additional imaging (e.g., contrast-enhanced magnetic resonance imaging [MRI] or ultrasound) was performed as clinically indicated (9). Baseline brain imaging and CSF analysis were routinely performed in patients with symptoms suggestive of CNS involvement or classified as high risk based on established criteria (20, 21).

Comprehensive medical record review captured clinical characteristics, laboratory data, treatment details, recurrence, and survival outcomes. Laboratory parameters included complete blood count (CBC), C-reactive protein (CRP), lymphocyte-to-monocyte ratio (LMR), platelet-to-lymphocyte ratio (PLR), albumin (ALB), β2-microglobulin (β2-MG), and lactate dehydrogenase (LDH) at diagnosis. Risk stratification followed NCCN-IPI criteria, classifying patients into low (0-1), low-intermediate (2–3), high-intermediate (4–5), and high-risk (≥6) categories (22). Additionally, patients were grouped into low, intermediate (22), and high CNS relapse risk categories according to CNS-IPI scores (8).

### 2.2 Cytokine measurements

Peripheral blood serum samples were collected from all patients at initial presentation. CSF was obtained via lumbar puncture when imaging confirmed safety, and was performed in CNS-symptomatic patients or those with high-risk features (e.g., intermediate/high CNS-IPI scores, testicular or breast involvement), following informed consent. Age-matched control patients hospitalized with non-neoplastic intracranial lesions during the same period were enrolled for comparative analysis; lumbar puncture eligibility was determined by neurologists.

Serum and CSF concentrations of IL-10 and IL-6 were measured using the IMMULITE/IMMULITE 1000 chemiluminescent immunoassay system (Siemens Healthineers). IL-6 was quantified using the Siemens LK6P1 assay kit and IL-10 using the LKXP1 assay kit. All assays were conducted by Sichuan West China Kang Shengda Medical Testing Co., Ltd.

### 2.3 Treatment regimen and evaluation

All patients received CD20 monoclonal antibody-based chemotherapy regimens, including R-CHOP or R-CHOP-like (rituximab, cyclophosphamide, doxorubicin/liposomal doxorubicin/etoposide, vincristine, prednisone), R plus dose-adjusted EPOCH (rituximab, etoposide, prednisone, vincristine, cyclophosphamide, doxorubicin), or BR (rituximab and bendamustine). Follow-up imaging was performed every 6 months for the first 2 years post-treatment and thereafter only as clinically indicated.

### 2.4 Definitions and endpoints

Cell of origin classification was determined using the Hans algorithm, categorizing tumors into germinal center B-cell (GCB) or non-GCB subtypes (23). Immunohistochemistry for MYC, BCL2, and BCL6 was performed. Dual-expressor lymphoma (DEL) was defined by MYC expression ≥40% and BCL2 expression ≥50% of tumor cells on immunohistochemistry (24). Progression-free survival (PFS) was calculated from diagnosis to progression, last follow-up, or death. Overall survival (OS) was measured from diagnosis to last follow-up or death from any cause.

### 2.5 Bioinformatics analysis of IL-10 and IL-6 in DLBCL

Publicly available gene expression datasets (GSE10846 and GSE87371) were obtained from the NCBI Gene Expression Omnibus (GEO) database. These datasets included RNA-sequencing data from tumor biopsy specimens of 414 and 223 treatment-naïve DLBCL patients, respectively, each with complete clinical follow-up data.

### 2.6 Statistical analysis

All statistical analyses were performed using SPSS version 26.0. GraphPad Prism 8.0 was used for data visualization. Continuous variables were presented as mean ± standard deviation (SD) for normally distributed data, or as median (range) for non-normally distributed data. Categorical variables were analyzed using the Chi-square or Fisher’s exact test; continuous variables were compared using the t-test or nonparametric equivalents. Receiver operating characteristic (ROC) curves were used to determine optimal cutoff values. Multivariate analyses were conducted using logistic regression. Kaplan-Meier curves and log-rank tests were employed to analyze PFS and OS. Cox regression was used for multivariate survival analysis. Correlation analyses were performed using Spearman’s rank correlation. A two-tailed *p*-value < 0.05 was considered statistically significant.

## 3 Results

### 3.1 Patients’ characteristics

This study included 56 newly diagnosed DLBCL patients without evidence of CNS involvement at baseline. Of these, 33 patients (58.9%) underwent PET/CT for systemic staging, while 23 (41.1%) received contrast-enhanced CT, with six patients in the CT group subsequently requiring brain or neck MRI. During follow-up, seven patients developed SCNSL. The median age was 65 years, and 41% (23/56) were female. Compared to those without SCNSL, patients who developed SCNSL exhibited significantly higher rates of B symptoms (57.1% vs. 12.2%, *p*=0.015), bone marrow involvement (71.4% vs. 14.3%, *p*=0.003), and lower BCL6 immunohistochemical (IHC) positivity rates (57.1% vs. 93.5%, *p*=0.025; **Table 1**). First-line treatment included R-CHOP or R-CHOP-like regimens in 49 patients, R-DA-EPOCH in 3 patients, and BR in 4 patients.

**Table 1:** General clinical characteristics of those with and without SCNSL.

Clinical Characteristics	Without SCNSL N=49 cases	With SCNSL N=7 cases	Statistical value	*p* value
Age	63.24 ± 12.85	61.43 ± 12.26	0.352	0.727
Sex			0.095	0.758
Male	57.1% (28/49)	71.4% (5/7)		
Female	42.9% (21/49)	28.6% (2/7)		
Ann Arbor stage			0.302	0.583
I-II	32.7% (16/49)	14.3% (1/7)		
III-IV	67.3% (33/49)	85.7% (6/7)		
ECOG score			0.000	1.000
0-1	57.1% (28/49)	57.1% (4/7)		
2-4	42.9% (21/49)	42.9% (3/7)		
B symptoms			NA	0.015*
No	87.8% (43/49)	42.9% (3/7)		
Yes	12.2% (6/49)	57.1% (4/7)		
NCCN-IPI			NA	0.406
Low	2.0% (1/49)	0.0% (0/7)		
Low/intermediate	28.6% (14/49)	0.0% (0/7)		
Intermediate/high	42.9% (21/49)	71.4% (5/7)		
High	26.5% (13/49)	28.6% (2/7)		
CNS-IPI			NA	0.136
Low	14.3% (7/49)	0.0% (0/7)		
Intermediate	42.9% (21/49)	85.7% (6/7)		
High	42.9% (21/49)	14.3% (1/7)		
Bone marrow involvement	14.3% (7/49)	71.4% (5/7)	8.727	0.003*
Kidney/adrenal gland involvement	14.3% (7/49)	14.3% (1/7)	NA	1.000
Extranodal involvement			NA	1.000
0-1	49.0% (24/49)	42.9% (3/7)		
≥ 2	51.0% (25/49)	57.1% (4/7)		
GCB type	22.9% (11/48)	14.3% (1/7)	0.001	0.979
BCL6 immunohistochemical positivity	93.5% (43/46)	57.1% (4/7)	NA	0.025*
C-MYC immunohistochemical positivity	93.3% (42/45)	80.0% (4/5)	NA	0.353
Dual expression	38.3% (18/47)	50.0% (3/6)	0.012	0.913
Pretreatment lymphocyte-to-monocyte ratio	2.34 (0.37-8.65)	1.45 (0.41-5.63)	1.697	0.090
Pretreatment platelet-to-lymphocyte ratio	190.23 (1.01-1652.50)	145.93 (5.83-323.81)	1.434	0.151
Pretreatment C-reactive protein	11.72 (0.42-194.42)	22.23 (1.35-198.75)	1.354	0.176
Pretreatment albumin (g/L)	38.91 ± 5.90	33.78 ± 8.97	1.466	0.188
Pretreatment β_2_-microglobulin (mg/L)	2.76 (1.18-8.15)	5.07 (3.10-5.94)	1.783	0.075
Pretreatment LDH (U/L)	389 (143-6693)	478 (198-4812)	1.016	0.310
Treatment regimens			NA	0.208
RCHOP/RCHOP like	89.8% (44/49)	10.2% (5/49)		
R-DA-EPOCH	66.7% (2/3)	33.3% (1/3)		
BR	75.0% (3/4)	25.0% (1/4)		

*, *p* < 0.05 was considered statistically significant. ECOG, eastern cooperative oncology group performance status. NCCN-IPI, national comprehensive cancer network international prognostic index. CNS-IPI, central nervous system international prognostic index. GCB, germinal center B cell. Dual expression was defined as MYC (≥40%) and BCL2 (≥50%) co-expression in tumor cells. LDH, lactate dehydrogenase. R-CHOP/R-CHOP like regimen, including rituximab, cyclophosphamide, doxorubicin/liposomal doxorubicin/etoposide, vincristine and prednisone. R-DA-EPOCH regimen, including rituximab, etoposide, prednisone, vincristine, cyclophosphamide and doxorubicin. BR regimen, including rituximab and bendamustine. NA, not available.

There were no statistically significant differences between patients with and without SCNSL in terms of age, sex, Ann Arbor stage, NCCN-IPI, CNS-IPI, cell of origin, or treatment regimen (*p* > 0.05). Similarly, baseline biochemical parameters—including CRP, LMR, PLR, ALB, β2-microglobulin, and LDH—did not differ significantly between the two groups (**Table 1**).

### 3.2 Risk factors for SCNSL

Serum IL-10 and IL-6 levels were available for 50 patients. At diagnosis, patients who developed SCNSL had significantly higher median serum IL-10 levels (30.80 pg/mL; range: 3.60–1000.00) compared to those who did not (3.00 pg/mL; range: 1.50–728.00) (Z = 3.396, *p* = 0.001; **Table 2**). Receiver operating characteristic (ROC) curve analysis identified 15.0 pg/mL as the optimal cutoff for serum IL-10; 71% of SCNSL cases had IL-10 levels ≥15.0 pg/mL. In contrast, serum IL-6 levels did not differ significantly between groups (6.43 vs. 5.38 pg/mL; Z = 0.253, *p* = 0.801). Analysis of serum IL-10/IL-6 ratios revealed significantly elevated values in SCNSL patients (median: 7.80; range: 0.81–69.67) versus non-SCNSL patients (median: 0.91; range: 0.01–22.69) (Z = 3.180, *p* = 0.001). ROC analysis determined 2.30 as the optimal cutoff for the IL-10/IL-6 ratio. Elevated ratios (≥2.30) were observed in 85.7% of SCNSL cases compared to 11.6% of those without SCNSL (*p* = 0.000; **Table 2**).

**Table 2:** Comparison of serum IL-10 and serum IL-6 in patients with and without SCNSL.

Clinical indicators	Without SCNSL N=43 cases	With SCNSL N=7 cases	Statistical value	*p* value
Serum IL-10 (pg/ml)	3.00 (1.50-728.00)	30.80 (3.60-1000)	3.396	0.001*
Serum IL- 6 (pg/ml)	5.38 (1.50-185.00)	6.43 (1.50-27.47)	0.253	0.801
Serum IL- 10/IL- 6	0.91 (0.01-22.69)	7.80 (0.81-69.67)	3.180	0.001*
Elevated serum IL-10 (≥15.0 pg/ml)	11.6% (5/43)	71.4% (5/7)	9.977	0.002*
Elevated serum IL-10/IL-6 (≥2.30)	11.6% (5/43)	85.7% (6/7)	15.180	0.000*

*, *p* < 0.05 was considered statistically significant. IL-10, interleukin-10. IL-6, interleukin-6.

Univariate analysis identified five variables significantly associated with SCNSL: B symptoms, bone marrow involvement, BCL6 IHC positivity, elevated serum IL-10, and elevated IL-10/IL-6 ratio (*p* < 0.05; **Tables 1**, **2**). These were included in a multivariate model, which identified the IL-10/IL-6 ratio as the sole independent predictor of SCNSL development (OR = 43.200; 95% CI: 4.269–437.158; *p* = 0.001).

**Figure 1:**
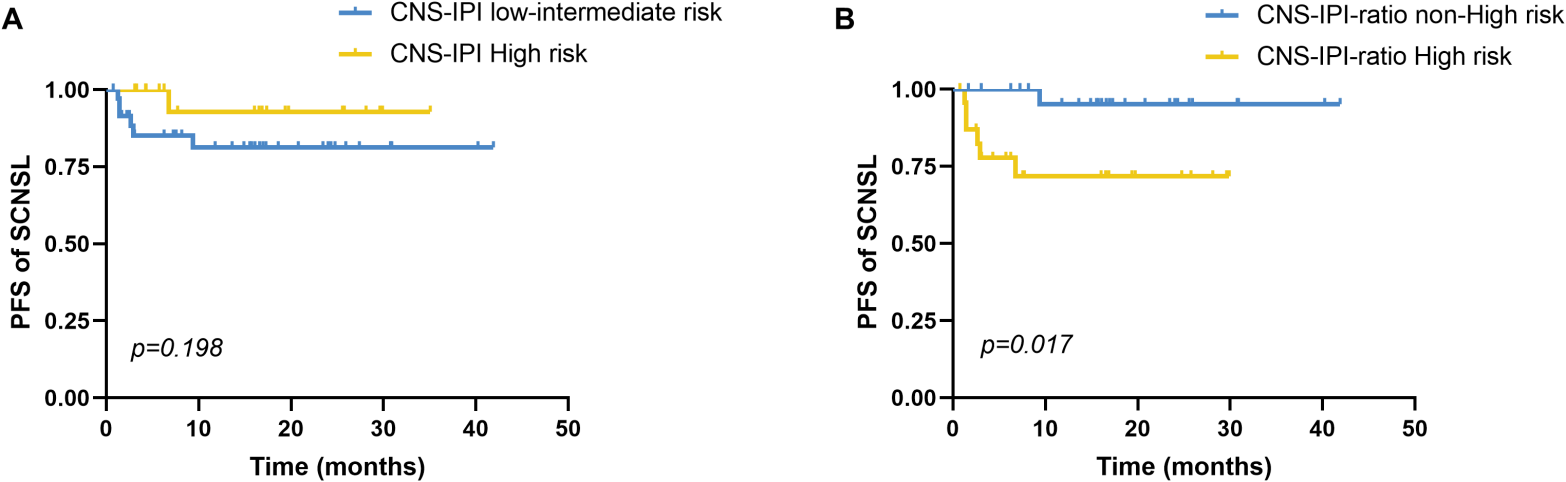
Progression-free survival to CNS relapse by CNS-IPI risk stratification. **(A)** Comparison of PFS until CNS relapse between the CNS-IPI low-intermediate risk (n=20) and high-risk (n=36) groups stratified by CNS-IPI, *p*=0.198. **(B)** The CNS-IPI-ratio scoring model integrates the CNS-IPI and elevated serum IL-10/IL-6 ratio. CNS-IPI low-, intermediate-, and high-risk levels scored 1, 2, and 3 points, respectively. An elevated serum IL-10/IL-6 ratio was assigned with 1 point. Based on the CNS-IPI-ratio, we divided all patients into the non-high-risk (scored 1-2) and high-risk (scored 3-4) groups. Comparison of PFS until CNS relapse between the CNS-IPI-ratio non-high-risk (n=24) and high-risk (n=26) groups stratified by the CNS-IPI-ratio, *p*=0.017. PFS, Progression-free survival. CNS, central nervous system, CNS-IPI, central nervous system international prognostic index. PFS was analyzed by the Kaplan-Meier method, and the difference between the groups was determined using the log-rank test.

Patients were stratified into CNS-IPI risk groups (low, intermediate, high), but the incidence of SCNSL did not differ significantly among these groups (*p* = 0.136). To enhance risk stratification, we integrated CNS-IPI scores with IL-10/IL-6 ratio status to construct a composite index (CNS-IPI-ratio). CNS-IPI scores of low, intermediate, and high were assigned 1, 2, and 3 points, respectively, with 1 additional point for IL-10/IL-6 ≥2.30. Patients were then classified as non–high risk (score 1–2) or high risk (score 3–4). Using this system, 85.7% (6/7) of SCNSL patients were categorized as high risk, significantly greater than the proportion among non-SCNSL patients (41.9%, 18/43; *p* = 0.045). Temporal analysis showed no significant difference in time to CNS relapse across original CNS-IPI risk groups (χ² = 1.654, *p* = 0.198; **Figure 1A**). In contrast, the CNS-IPI-ratio scoring system demonstrated improved prognostic discrimination, with the high-risk group exhibiting a significantly shorter median time to CNS relapse (22.32 vs. 40.35 months; χ² = 5.680, *p* = 0.017; **Figure 1B**).

### 3.3 Prognostic value of the serum IL-10/IL-6 ratio

**Figure 2:**
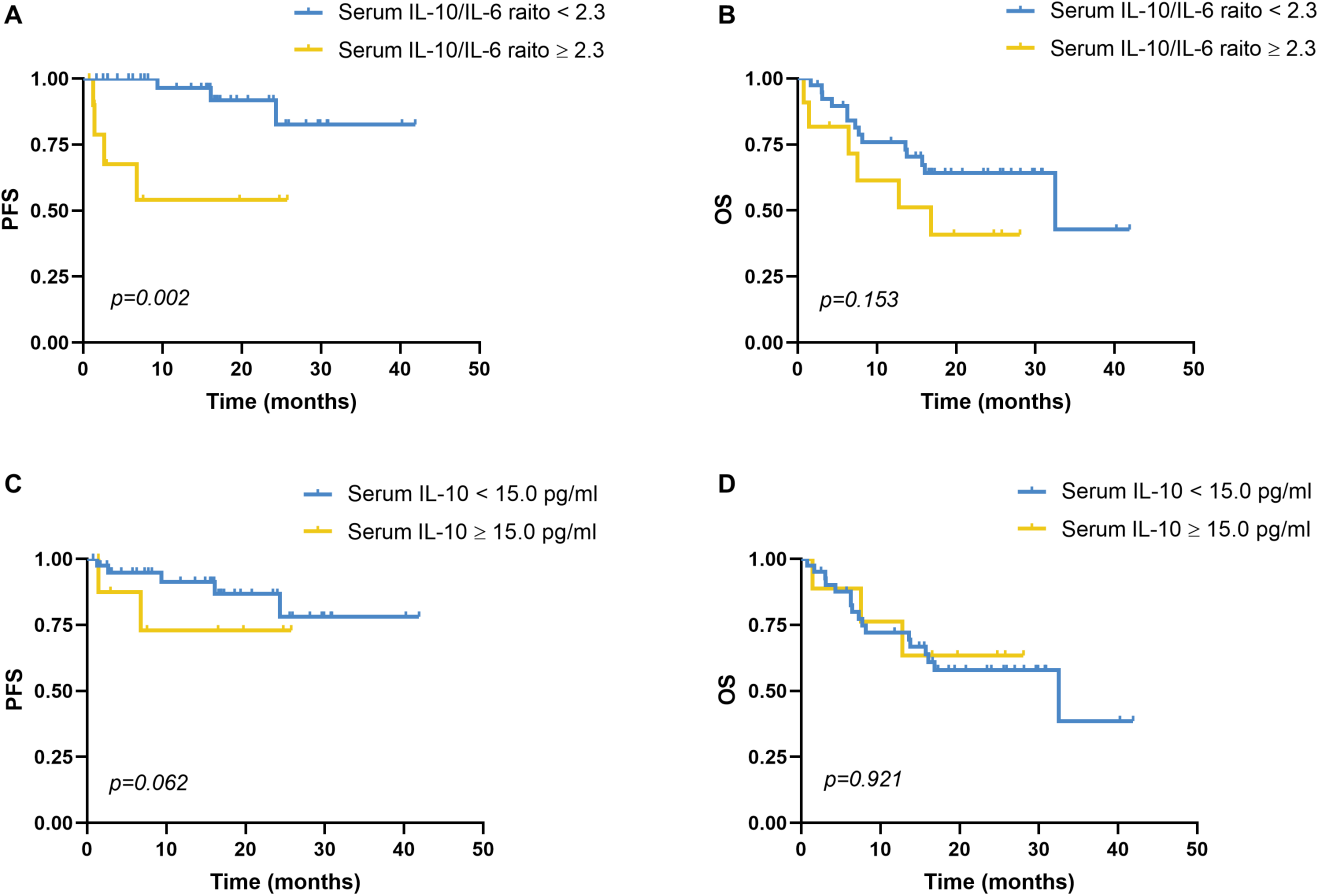
Progression-free survival and overall survival analyses of serum IL-10 and serum IL-10/IL-6. **(A)** PFS analysis in patients with a reduced serum IL-10/IL-6 ratio (<2.30, n=39) and an elevated serum IL-10/IL-6 ratio (≥2.30, n=11), *p*=0.002. Using disease recurrence as the endpoint, ROC curve analysis revealed that the optimal cutoff value of the serum IL-10/IL-6 ratio was 2.3. **(B)** OS analysis in patients with a reduced serum IL-10/IL-6 ratio (<2.30, n=39) and an elevated serum IL-10/IL-6 ratio (≥2.30, n=11), *p*=0.153. **(C)** PFS analysis in patients with a reduced serum IL-10 level (<15.0 pg/mL, n=41) and an elevated serum IL-10 level (≥15.0 pg/mL, n=9), *p*=0.062. With disease recurrence as the endpoint, ROC curve analysis identified the optimal cutoff value of the serum IL-10 level was 15.0 pg/mL. **(D)** OS analysis in patients with a reduced serum IL-10 level (<15.0 pg/mL, n=41) and an elevated serum IL-10 level (≥15.0 pg/mL, n=9), *p*=0.921. PFS, progression-free survival; OS, overall survival; IL-10, interleukin-10; IL-6, interleukin-6. PFS and OS were analyzed by the Kaplan-Meier method, and the difference between the groups was determined using the log-rank test.

PFS was significantly shorter in patients with elevated IL-10/IL-6 ratios (median: 15.42 months; 95% CI: 7.82–23.01) compared to those with lower ratios (median: 37.94 months; 95% CI: 33.75–42.14; χ² = 9.678, *p* = 0.002; **Figure 2A**). However, overall survival (OS) did not differ significantly between the two groups (16.10 vs. 27.93 months; χ² = 2.042, *p* = 0.153; **Figure 2B**). Elevated IL-10 alone was not significantly associated with either PFS (*p* = 0.062) or OS (*p* = 0.921; **Figures 2C, D**).

**Figure 3:**
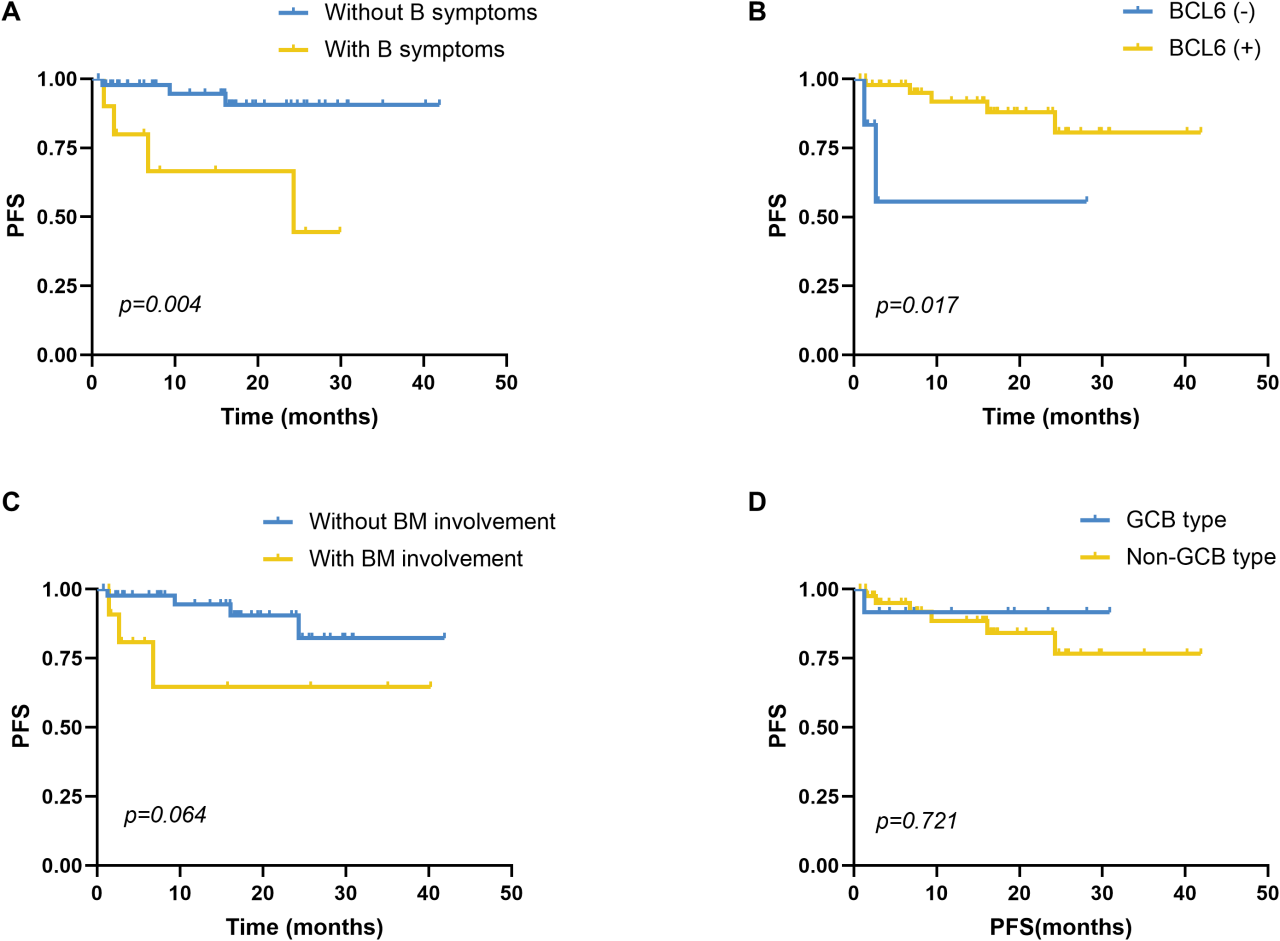
Survival analysis of PFS in DLBCL patients. **(A)** Comparison of PFS between patients with (n=10) and without (n=46) symptoms, *p*=0.004. **(B)** Comparison of PFS between patients with BCL6 IHC positivity (n=47) and negativity (n=6), *p*=0.017. **(C)** Comparison of PFS between patients with (n=12) and without BM involvement (n=44), *p*=0.064. **(D)** Comparison of PFS between patients with the GCB type (n=12) and non-GCB type (n=43), *p*=0.721. GCB and non-GCB subtypes based on the Hans algorithm. PFS, progression-free survival; DLBCL, diffuse large B-cell lymphoma; BM, bone marrow, GCB, germinal center B cell; non-GCB, non-germinal center B cell. PFS was analyzed using the Kaplan-Meier method, and the difference between the groups was determined using the log-rank test.

Univariate analysis showed that PFS was significantly associated with B symptoms and BCL6 IHC positivity (**Figures 3A, B**). Other variables—including bone marrow involvement, C-MYC expression, GCB subtype, MYC/BCL2 co-expression, and six-course treatment—did not significantly affect PFS (**Figures 3C, D**; *p* > 0.05). Multivariate analysis incorporating B symptoms, BCL6 positivity, and IL-10/IL-6 ratio ≥2.30 identified the IL-10/IL-6 ratio as the only independent predictor of shorter PFS (HR = 7.300; 95% CI: 1.615–33.006; *p* = 0.010).

**Figure 4:**
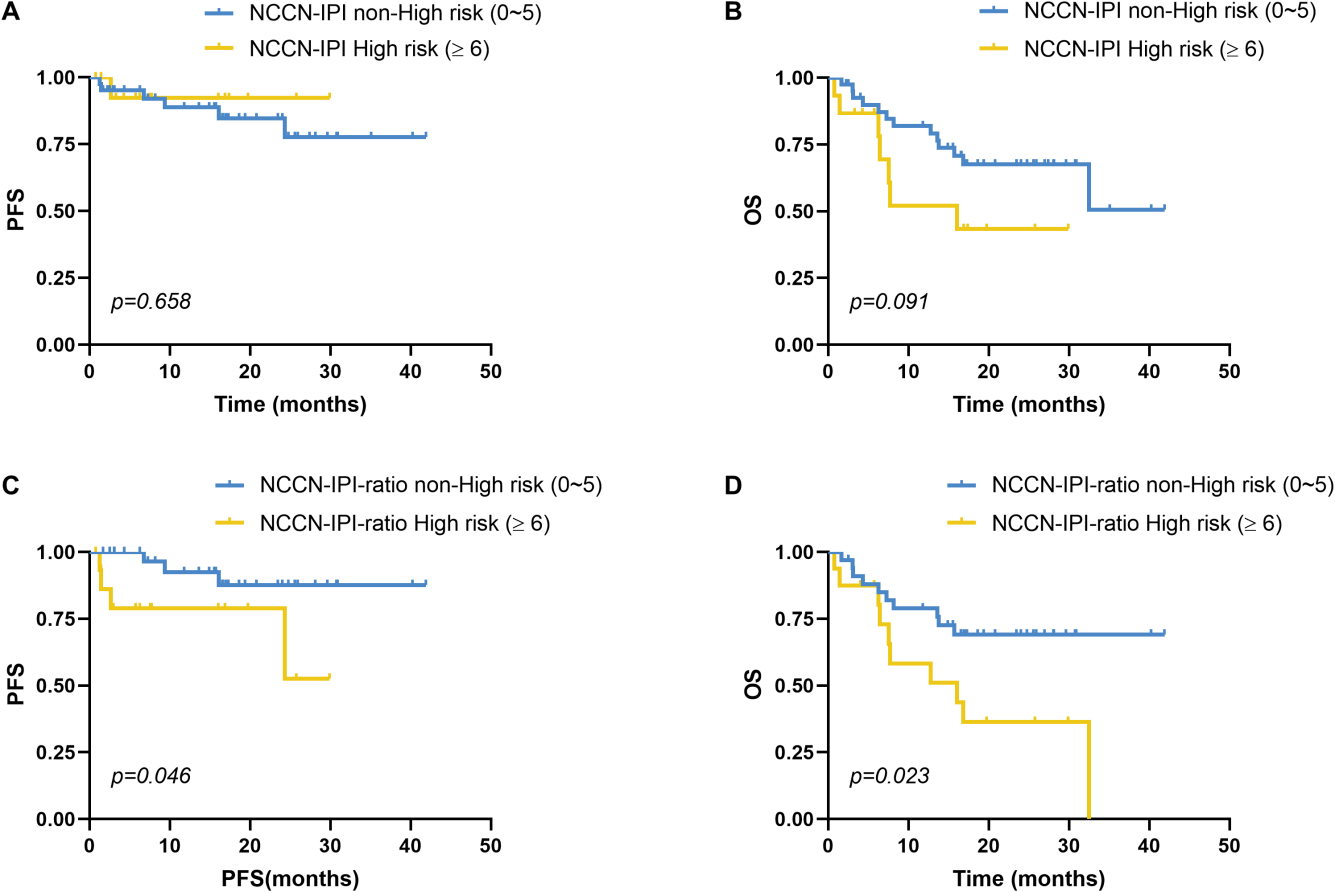
Impact of NCCN-IPI risk stratification on survival in DLBCL. **(A)** PFS and **(B)** OS comparisons between the NCCN-IPI high-risk (n=15) and non-high-risk (n=41) groups showed no statistically significant differences (PFS: *p*=0.658; OS: *p*=0.091). **(C-D)** Novel NCCN-IPI-ratio scoring system was created by adding 1 point to standard NCCN-IPI scores for patients with elevated serum IL-10/IL-6 ratios (≥2.30 pg/mL). **(C)** PFS and **(D)** OS comparisons between the NCCN-IPI-ratio high-risk (n=16) and non-high-risk (n=34) groups showed statistically significant differences (PFS: *p*=0.046; OS: *p*=0.023). Stratification thresholds followed standard NCCN-IPI criteria (high risk: ≥6) and were equivalently applied to the NCCN-IPI-ratio. PFS and OS were analyzed using the Kaplan-Meier method, and the difference between the groups was determined using the log-rank test.

Patients with NCCN-IPI scores of 0–5 were classified as the non–high-risk group, whereas those with scores ≥6 comprised the high-risk group. The high-risk group had a median PFS of 27.81 months (95% CI: 23.86–31.75), compared to 35.50 months (95% CI: 30.85–40.14) in the non–high-risk group; however, this difference was not statistically significant (χ²=0.196, *p*=0.658; **Figure 4A**). Similarly, the median OS was 16.92 months (95% CI: 10.29–23.55) in the high-risk group versus 29.69 months (95% CI: 24.30–35.08) in the non–high-risk group, with no significant difference (χ²=2.848, *p*=0.091; **Figure 4B**).

We next developed a novel prognostic model, termed the NCCN-IPI-ratio, by integrating the serum IL-10/IL-6 ratio into the traditional NCCN-IPI framework. Specifically, patients with elevated IL-10/IL-6 ratios (≥2.30 pg/mL) received an additional 1 point on the NCCN-IPI. This modified score preserved the original risk stratification scheme, defining non–high-risk (0–5 points) and high-risk (≥6 points) categories. The NCCN-IPI-ratio yielded clinically meaningful prognostic discrimination. High-risk patients (≥6 points) had a significantly shorter median PFS (22.53 months, 95% CI: 16.50–28.56) compared to non–high-risk patients (38.14 months, 95% CI: 34.13–42.14), with the difference reaching statistical significance (χ²=3.989, *p*=0.046; **Figure 4C**). Similarly, median OS was significantly shorter in the high-risk group (17.36 months, 95% CI: 10.66–24.06) versus the non–high-risk group (31.39 months, 95% CI: 25.90–36.87; χ²=5.135, *p*=0.023; **Figure 4D**).

### 3.4 IL-10 and IL-6 mRNA expression in GEO datasets

Given the limited sample size in our primary cohort, we further validated these findings using publicly available GEO datasets (GSE87371 and GSE10846) to analyze IL-10 and IL-6 mRNA expression profiles from RNA-seq data.

In the prospective multicenter LYSA cohort (GSE87371, n=223), IL-10/IL-6 mRNA expression ratios were significantly associated with clinical outcomes. ROC analysis identified an optimal cutoff of 1.27 for predicting disease progression (AUC=0.572). Patients with ratios <1.27 had significantly longer mean PFS (56.52 months, 95% CI: 50.66–62.38) than those with ratios ≥1.27 (52.48 months, 95% CI: 45.71–59.26; log-rank χ²=5.001, *p*=0.025; **Supplementary Figure 1A**). For OS, the optimal cutoff was 1.65 (AUC=0.560). Patients with ratios <1.65 had superior mean OS (64.01 months, 95% CI: 58.46–69.56) compared to those with ratios ≥1.65 (50.53 months, 95% CI: 43.78–57.28; χ²=4.678, *p*=0.031; **Supplementary Figure 1B**). High-risk IPI scores (≥4) were associated with significantly worse PFS (χ²=19.442, *p*<0.001). Incorporating the IL-10/IL-6 ratio (≥1.27 scored as 1 point) into the IPI model, patients with IPI-ratio scores ≥5 had significantly poorer PFS than those with scores <5 (χ²=22.240, *p*<0.001; **Supplementary Figure 1C**), with improved prognostic discrimination (χ² increase from 19.442 to 22.240). Similarly, for OS, patients with high IPI scores (≥4) had significantly worse outcomes (χ²=33.378, *p*<0.001). The integrated IPI-ratio model (ratio ≥1.65 scored as 1 point) further enhanced OS stratification, with high-risk patients (score ≥5) showing significantly shorter survival (χ²=52.641, *p*<0.001; **Supplementary Figure 1D**), and improved discriminatory power (χ² increase from 33.378 to 52.641).

In the retrospective cohort (GSE10846, n=414), including 181 CHOP-treated and 233 R-CHOP–treated patients, ROC analysis yielded an optimal IL-10/IL-6 cutoff of 1.0 for OS prediction (AUC=0.483). Although the survival difference did not reach statistical significance (χ²=1.124, *p*=0.289), patients with ratios <1.0 demonstrated a numerically longer mean OS (11.42 years, 95% CI: 9.66–13.19) than those with ratios ≥1.0 (5.78 years, 95% CI: 4.94–6.63), suggesting a potential prognostic trend.

### 3.5 CSF IL-10 and CSF IL-6

**Figure 5:**
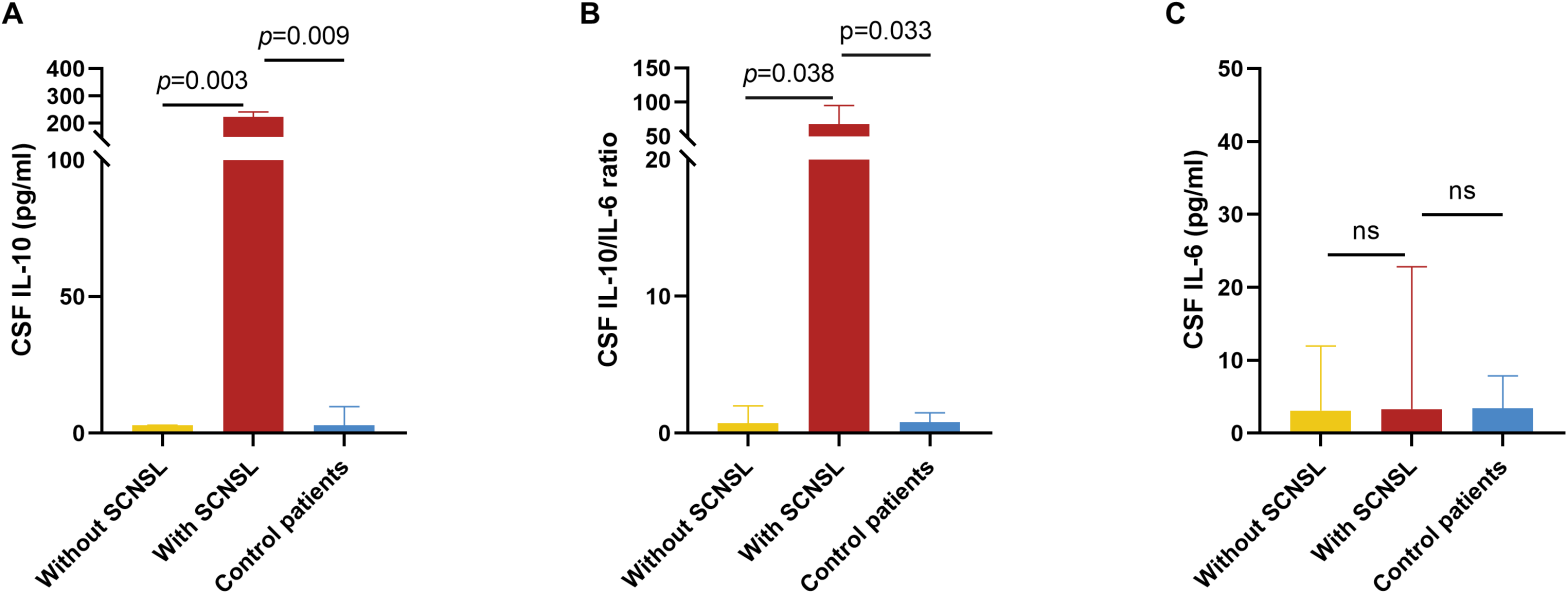
CSF IL-10 and CSF IL-6 levels in patients with and without SCNSL. For a comparative analysis, we concurrently evaluated 9 age-matched control patients hospitalized with non-neoplastic intracranial lesions during the same study period. **(A)** Concentrations of CSF IL-10 in patients with SCNSL, patients without SCNSL and control patients. n=3, 15, and 9, respectively. **(B)** Ratio of CSF IL-10/IL-6 in patients with SCNSL, patients without SCNSL, and control patients. n=3, 15, and 9, respectively. **(C)** Concentrations of CSF IL-6 in patients with SCNSL, patients without SCNSL and control patients. n=3, 15, and 9, respectively. ns, not significant values are indicated. SCNSL, secondary central nervous system lymphoma; CSF, cerebrospinal fluid; IL-10, interleukin-10; IL-6, interleukin-6.

Among the enrolled DLBCL patients, CSF IL-10 and IL-6 levels were measured in 18 cases, including 3 who later developed SCNSL. For comparative purposes, we also evaluated 9 age-matched controls hospitalized with non-neoplastic intracranial conditions. CSF cytokine concentrations are detailed in **Table 3**. Patients with SCNSL exhibited significantly elevated CSF IL-10 levels compared to both non-SCNSL patients (median: 224.0 vs 3.0 pg/mL; Z=2.963, *p*=0.003) and controls (224.0 vs 3.0 pg/mL; Z=2.608, *p*=0.009; **Figure 5A**). Similarly, the CSF IL-10/IL-6 ratio was markedly higher in SCNSL patients relative to both non-SCNSL patients (median: 67.88 vs 0.74; Z=2.074, *p*=0.038) and controls (67.88 vs 0.79; Z=2.126, *p*=0.033; **Figure 5B**). However, these findings should be interpreted cautiously given the limited number of SCNSL cases (n=3), which constrains cutoff reliability and underscores the need for validation in larger cohorts. Notably, no significant difference in CSF IL-6 levels was observed between SCNSL and non-SCNSL patients (3.30 vs 3.09 pg/mL; Z=0.889, *p*=0.374; **Figure 5C**).

**Table 3:** Comparison of CSF IL-10 and CSF IL-6 in those with and without SCNSL and concurrent hospitalized control patients.

Clinical indicators	Without SCNSL N=15 cases	With SCNSL N=3 cases	Control patients N=9 cases
CSF IL-10 (pg/ml)	3.0 (1.5-3) ^a^	224.0 (27.3-241.51) ^ab^	3.0 (1.5-9.73) ^b^
CSF IL-6 (pg/ml)	3.09 (1.50-11.96)	3.30 (2.53-22.80)	3.41 (2.01-7.85)
CSF IL-10/IL- 6	0.74 (0.13-2.00) ^c^	67.88 (1.20-95.46) ^cd^	0.79 (0.35-1.49) ^d^

CSF, cerebrospinal fluid. IL-10, interleukin-10. IL-6, interleukin-6. SCNSL, secondary central nervous system lymphoma. ^a^ or ^c^, comparisons between patients with and without SCNSL, *p*<0.05. ^b^ or ^d^, comparisons between patients with SCNSL and patients concurrently hospitalized with non-neoplasitc intracranial lesions, *p*<0.05.

### 3.6 Correlation of serum IL-6 and IL-10 with CSF IL-6 and IL-10

**Figure 6:**
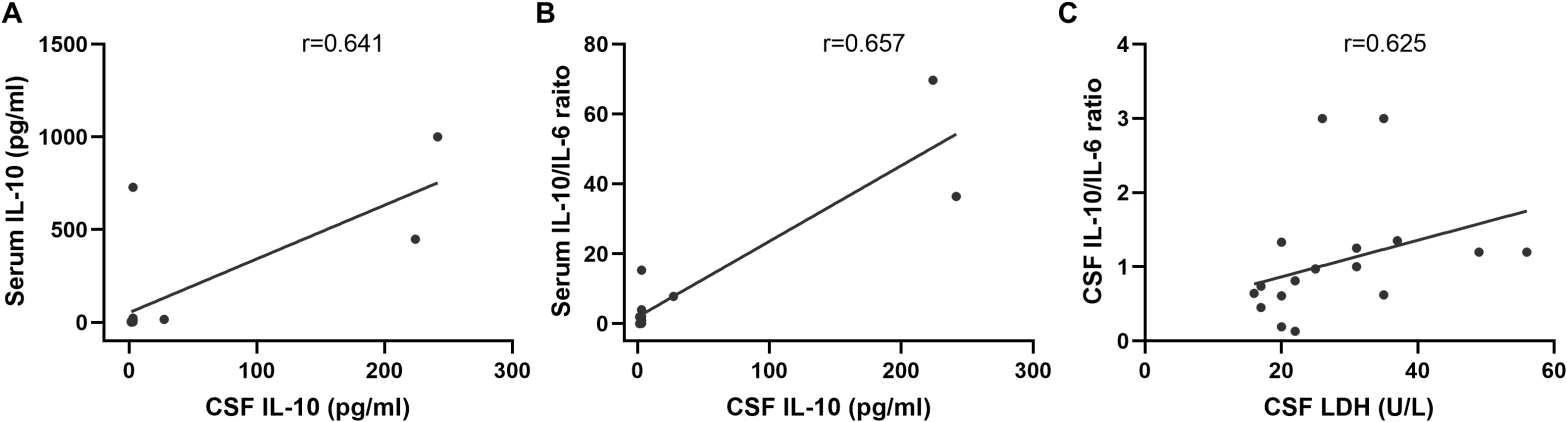
Correlation of serum IL-6 and IL-10 with CSF IL-6 and IL-10 levels. **(A)** Significant correlation between serum IL-10 and CSF IL-10 (r=0.641, n=14, *p*=0.014). **(B)** Serum IL-10/IL-6 ratio showing a significant correlation with CSF IL-10 concentrations (r=0.657, n=14, *p*=0.011). **(C)** CSF IL-10/IL-6 ratio exhibiting a notable correlation with LDH levels (r=0.625, n=18, *p*=0.006).

Paired serum and CSF IL-10 measurements were available for 15 patients. A significant positive correlation was observed between serum and CSF IL-10 levels (r = 0.641, *p* = 0.014; **Figure 6A**). Similarly, the serum IL-10/IL-6 ratio was significantly correlated with CSF IL-10 concentrations (r = 0.657, *p* = 0.011; **Figure 6B**). In a separate cohort of 18 patients with matched CSF IL-10, IL-6, and LDH data, the CSF IL-10/IL-6 ratio was significantly correlated with CSF LDH levels (r = 0.625, *p* = 0.006; **Figure 6C**). However, neither CSF IL-10 (*p* = 0.106) nor CSF IL-6 alone (*p* = 0.193) showed a significant association with CSF LDH.

## 4 Discussion

SCNSL represents a devastating complication in DLBCL with uniformly poor outcomes. The CNS-IPI does not account for the biological heterogeneity of DLBCL (2), limiting its ability to effectively stratify patients at risk of CNS involvement. Previous reports have linked elevated serum levels of IL-6 and IL-10 to adverse prognosis in DLBCL (25, 26). In the present study, we investigated the clinical utility of serum IL-6, IL-10, and the IL-10/IL-6 ratio, analyzing their associations with disease progression and SCNSL development. Our findings demonstrate that the serum IL-10/IL-6 ratio functions as an independent risk factor for both disease progression and CNS relapse. Moreover, incorporating this biomarker into CNS-IPI and NCCN-IPI models significantly improved their ability to identify high-risk patients.

Chronic inflammation creates a microenvironment conducive to tumor initiation, promotion, progression, metastasis, and resistance to chemotherapy, primarily through the activity of inflammatory cells, cytokines, chemokines, and other mediators. Key cytokines within this milieu include IL-6, IL-10, among others (27). Elevated serum IL-6 levels have been consistently reported in patients with DLBCL compared to healthy controls (28, 29). Dlouhy et al. (30) demonstrated that higher serum IL-6 levels were significantly associated with shorter PFS in DLBCL. More recently, Bao et al. (10) showed that increased IL-6 not only correlates with adverse clinical features and PFS but also functions as an independent prognostic marker for relapse and OS. Compared with patients exhibiting normal serum IL-6 levels, those with elevated IL-6 were more likely to exhibit partial or no response to therapy.

Similarly, several studies have reported that elevated serum IL-10 at diagnosis is associated with unfavorable disease features and poor clinical outcomes (31–33). In comparison with healthy controls, DLBCL patients demonstrated significantly higher serum IL-10 levels (*p* < 0.001). Patients with IL-10 concentrations ≥5.0 pg/mL experienced significantly shorter PFS and OS (*p* < 0.001) (10). Yi et al. (1) investigated the association between pretreatment serum cytokines and the occurrence of SCNSL in DLBCL. Their findings revealed that patients with elevated serum IL-10 levels were more likely to develop SCNSL and experienced earlier CNS involvement. Moreover, CNS infections or inflammatory conditions can elevate IL-10 levels in CSF, often accompanied by a reduced IL-10/IL-6 ratio (34, 35). This may reflect a compensatory anti-inflammatory response by immune cells in reaction to pro-inflammatory cytokines such as IL-6 (12, 36). Therefore, in this study, we also evaluated the IL-10/IL-6 ratio in both CSF and serum to determine its utility in identifying patients at high risk for SCNSL.

Our study explored the prognostic significance of serum IL-10 and IL-6 in the development of SCNSL and uncovered several clinically relevant insights. Although both cytokines were assessed, only serum IL-10 levels were significantly elevated in SCNSL patients (30.80 vs. 3.00 pg/mL, *p* = 0.001), suggesting a predominant role in CNS tropism. Importantly, the serum IL-10/IL-6 ratio emerged as a particularly powerful biomarker, demonstrating an 8.6-fold increase in SCNSL patients (7.80 vs. 0.91 pg/mL, *p* = 0.001). This ratio may better reflect the immunosuppressive tumor microenvironment than absolute cytokine levels alone, as the anti-inflammatory effects of IL-10 may dominate when disproportionately elevated relative to IL-6. Multivariate analysis confirmed that a serum IL-10/IL-6 ratio ≥2.3 was the only independent predictor of both poor PFS (HR = 7.30, *p* = 0.010) and SCNSL development (odds ratio [OR] = 43.20, *p* = 0.001). The strength of these associations highlights the ratio’s potential clinical relevance. Supporting this, patients with elevated ratios had significantly shorter median PFS (15.42 vs. 37.94 months, *p* = 0.002), representing a clinically meaningful 22.5-month difference. These findings support a novel paradigm in which the prognostic impact of IL-10 in DLBCL is modulated not solely by its absolute level, but by its balance relative to IL-6.

The Central Nervous System International Prognostic Index (CNS-IPI) is currently the most widely used tool for predicting CNS involvement in DLBCL. In our cohort of 56 patients, CNS-IPI stratification—though limited by a median follow-up of 23.5 months (range: 0.7–41.9 months) and sample size—did not demonstrate statistically significant differences in SCNSL incidence among the low-, intermediate-, and high-risk groups (*p* = 0.136). Similarly, no significant difference in time to CNS relapse was observed between high-risk and non–high-risk groups. To improve risk prediction, we developed a modified scoring system, termed CNS-IPI-ratio, which integrates the serum IL-10/IL-6 ratio with the CNS-IPI score. Risk levels in the original CNS-IPI were assigned 1, 2, and 3 points for low, intermediate, and high risk, respectively; an additional point was assigned for a serum IL-10/IL-6 ratio ≥2.30. Patients scoring 1–2 points were classified as non–high-risk, while those scoring 3–4 points were categorized as high-risk. Our stratification approach yielded two key clinical findings. First, the CNS-IPI-ratio high-risk group identified significantly more SCNSL cases than the non–high-risk group (85.7% vs. 41.9%, *p* = 0.045), outperforming conventional models in discriminative capacity. Second, high-risk patients developed CNS relapse nearly 18 months earlier than non–high-risk patients (median 22.32 vs. 40.35 months, *p* = 0.017), indicating that CNS-IPI-ratio may predict both the likelihood and timing of CNS progression. The National Comprehensive Cancer Network International Prognostic Index (NCCN-IPI) remains the standard prognostic model for DLBCL in the rituximab era (22). However, this model relies solely on clinical parameters and excludes biological markers. In our analysis, the NCCN-IPI alone showed a non-significant trend toward PFS and OS differences between high-risk and non–high-risk groups. After incorporating the serum IL-10/IL-6 ratio into the NCCN-IPI, the modified scoring system yielded statistically significant differences in both PFS and OS. These findings suggest that integrating the serum IL-10/IL-6 ratio significantly enhances the prognostic accuracy of the NCCN-IPI.

Tumor RNA-seq analyses across multiple DLBCL cohorts consistently demonstrated that elevated IL-10/IL-6 expression ratios were associated with poorer clinical outcomes. Notably, an integrated IPI-ratio model provided superior prognostic discrimination compared to the conventional IPI alone. Although tumor mRNA expression data from GEO cohorts support the biological relevance of our serum biomarker findings, they serve a complementary rather than substitutive role. These transcriptomic analyses reinforce the mechanistic basis and cross-cohort consistency of IL-10/IL-6 dysregulation, yet the clinical utility of the serum IL-10/IL-6 ratio must be directly validated in our ongoing prospective study, as serum protein levels in an independent cohort remain the gold standard for biomarker evaluation. The consistent demonstration of IL-10/IL-6 imbalance in both the peripheral circulation (serum) and tumor microenvironment (RNA-seq) underscores the biological significance of this cytokine axis in DLBCL pathogenesis. While incorporating the IL-10/IL-6 ratio into existing clinical models enhances risk stratification, these preliminary findings warrant prospective validation in multicenter cohorts before clinical implementation.

The diagnostic value of elevated CSF IL-10 remains debated. Ikeguchi et al. (37) reported that although median CSF IL-10 concentrations were significantly higher in patients with CNS lymphoma (including PCNSL and SCNSL) compared to those with inflammatory demyelinating CNS diseases (*p* < 0.01), multivariate analysis indicated that CSF IL-10 lacked independent predictive value. In contrast, numerous studies have supported the utility of elevated CSF IL-10 in distinguishing PCNSL from other brain tumors, neuroinflammatory conditions, and systemic DLBCL (38–41). Notably, increased CSF IL-10 has been proposed as a potential biomarker for CNS lymphoma, including SCNSL (14, 15). On the other hand, IL-6 is frequently detectable in CSF during viral, bacterial, and fungal infections, as well as CNS inflammatory diseases—especially bacterial meningitis (42, 43). Patients with CNS inflammatory disorders had significantly higher CSF IL-6 concentrations than those with PCNSL (p = 0.032) (41). Most studies evaluating the CSF IL-10/IL-6 ratio have focused on PCNSL, with this ratio frequently serving as a diagnostic marker. For example, a CSF IL-10/IL-6 ratio >1 was detected in 75% (58/77) of PCNSL cases (44), and a similar threshold in vitreous or atrial fluid strongly suggested primary vitreoretinal lymphoma (PVRL) (45). Additionally, studies by Song (12) and Li et al. (46) reported that the CSF IL-10/IL-6 ratio could aid in distinguishing PCNSL from systemic non-Hodgkin lymphoma. When comparing the CSF IL-6/IL-10 ratio between CNS inflammatory disease and PCNSL, the ratio was significantly higher in the former (*p* < 0.001) (41). In our study, CSF IL-10/IL-6 ratios were significantly elevated in SCNSL patients relative to both non-SCNSL DLBCL patients and healthy controls. However, given the small number of SCNSL patients with available CSF samples (n = 3), these findings should be interpreted as preliminary and hypothesis-generating rather than definitive. While these observations are biologically plausible and consistent with prior PCNSL research, larger, multicenter prospective studies are needed to establish their diagnostic validity.

Regarding the serum IL-10/IL-6 ratio, our findings identified it as an independent risk factor for both progression-free survival (PFS) and SCNSL development in DLBCL. Serum IL-10 levels have previously been shown to reflect systemic tumor burden (1). Additionally, IL-10 plays a pivotal role in the JAK/STAT signaling pathway and can induce PD-L1 expression, facilitating immune evasion by tumor cells (1, 47). IL-10 is also implicated in blood-brain barrier dysfunction, promoting CNS infiltration by malignant cells (1, 16, 48). In contrast, IL-6—being a pleiotropic cytokine—is elevated in nearly all infectious or inflammatory conditions (42). Therefore, an elevated IL-10/IL-6 serum ratio may help exclude patients with underlying systemic infection or inflammation, thereby improving specificity for tumor-associated cytokine imbalances. A higher IL-10/IL-6 ratio at initial diagnosis may thus serve as a potential biomarker for identifying DLBCL patients at high risk for SCNSL. However, some studies have reported contradictory findings. Zou (49) observed no significant difference in serum IL-10/IL-6 ratios between DLBCL patients with and without SCNSL. Similarly, Geng et al. (38) found no statistically significant difference in this ratio between PCNSL patients and controls (patients with intracranial metastatic tumors or gliomas). These discrepancies may be attributable to differences in sample size, disease stage, or clinical characteristics across cohorts. Given the collective evidence on serum and CSF IL-10/IL-6 dynamics in DLBCL, SCNSL, and PCNSL, we systematically summarized current literature findings in **Table 4**. Distinct from prior PCNSL-focused studies (12–15), our data suggest that serum IL-10/IL-6 ratios can predict CNS relapse risk in asymptomatic DLBCL patients, potentially enabling preemptive therapeutic strategies. Importantly, unlike CSF-based diagnostics, our serum-based approach is minimally invasive and requires no additional sampling. The strong correlation observed between serum and CSF ratios further suggests that systemic immune dysregulation may precede CNS involvement, offering a critical window for early detection.

**Table 4:** Clinical Significance of Serum and CSF IL-10 and IL-6 in DLBCL, SCNSL, and PCNSL.

Biomarker	DLBCL		SCNSL		PCNSL	
	Prognostic/diagnostic significance	References	Prognostic/diagnostic significance	References	Prognostic/diagnostic significance	References
Serum IL-10	Elevated serum IL-10 (the thresholds including: ≥5.0 pg/mL, ≥20.2 pg/mL, and ≥26 pg/mL) correlated with adverse clinical features (such as higher level of LDH and β2-MG), poor prognosis (PFS, OS, and response to therapy et al.), and might serve as a prognostic factor for relapse and survival.	Yi et al. (2020) (1); Bao et al. (2023) (10); Zhang et al. (2021) (11); Načinović-Duletić et al. (2008) (25); Xie et al. (2024) (28); Lech-Maranda et al. (2010) (31); Pauly et al. (2016) (32); Nguyen-Them et al. (2023) (39); Gupta et al. (2012) (16); Béguelin et al. (2015) (48)	Patients with increased level of serum IL-10 had shorter time to CNS invasion, and might predict the risk of SCNSL in patients with DLBCL. And when 0.2 < CSF IL-10/plasma IL-10 ratio < 5.0, this ratio also could be a reliable biomarker to diagnose SCNSL.	Yi et al. (2020) (1); Zou et al. (2023) (49);	Compared to group with other brain tumors, patients with PCNSL had higher serum IL-10 level.	Geng et al. (2021) (38);
Serum IL-6	Higher levels of serum IL-6 (the cutoff values including: ≥4.5 pg/mL, ≥20 pg/mL, and ≥28 pg/mL) indicated adverse clinical features (higher level of LDH, β2-MG), correlated with poor PFS, and might serve as a prognostic factor for relapse and survival.	Bao et al. (2023) (10); Zhang et al. (2021) (11); Načinović-Duletić et al. (2008) (25); Liévin et al. (2024) (26); Xie et al. (2024) (28); Dlouhy et al. (2017) (30); Pauly et al. (2016) (32);	NA	NA	NA	NA
Serum IL-10/IL-6	NA	NA	This ratio had no statistical difference between patients with and without SCNSL.	Zou et al. (2023) (49);	The ratio had no statistical difference between PCNSL patients and the other brain tumors group.	Geng et al. (2021) (38);
CSF IL-10	NA	NA	Increased CSF IL-10 level (≥7.82 pg/mL) correlated with shorter PFS, and had the potential to serve as a diagnostic biomarker for SCNSL.	Gu et al. (2023) (14) Rubenstein et al. (2013) (15);	Increased CSF IL-10 level (cutoff values including: ≥0.43 pg/mL, ≥2 pg/mL, ≥4 pg/mL, ≥8.2 pg/mL, ≥8.3 pg/mL, ≥10.13 pg/mL, and ≥16.15 pg/mL) indicated poor PFS, facilitated discriminating PCNSL from systemic NHL and other CNS diseases (infection/inflammation, or other malignant tumors in CNS).	Shao et al. (2020) (13); Rubenstein et al. (2013) (15); Šúri et al. (2025) (34); Westrhenen et al. (2018) (35); Song et al. (2016) (12); Geng et al. (2021) (38); Nguyen-Them et al. (2023) (39); Ferreri et al. (2021) (40); Ungureanu et al. (2021) (41); Armand et al. (2019) (44); Li et al. (2023) (46);
CSF IL-6	NA	NA	Elevated CSF IL-6 concentration (≥10.13 pg/mL) might be a potential diagnostic biomarker for SCNSL.	Gu et al. (2023) (14);	CSF IL-6 concentration increased in PCNSL patients, but it was lower than patients with CNS inflammatory disease.	Ungureanu et al. (2021) (41);
CSF IL-10/IL-6	NA	NA	NA	NA	Elevated CSF IL-10/IL-6 ratio (the thresholds including: ≥0.21, ≥0.72, ≥1, and ≥1.6) could identify PCNSL patients from infection of CNS, other brain tumors, and systemic NHL patients.	Shao et al. (2020) (13); Šúri et al. (2025) (34); Westrhenen et al. (2018) (35); Song et al. (2016) (12); Geng et al. (2021) (38); Ferreri et al. (2021) (40); Ungureanu et al. (2021) (41); Armand et al. (2019) (44); Huang et al. (2024) (45); Li et al. (2023) (46);

CSF, cerebrospinal fluid. IL-10, interleukin-10. IL-6, interleukin-6. DLBCL, diffuse large B-cell lymphoma. SCNSL, secondary central nervous system lymphoma. PCNSL, primary central nervous system lymphoma. NHL, non-Hodgkin lymphoma. CNS, central nervous system. LDH, lactate dehydrogenase. β2-MG, β2-microglobulin. PFS, progression-free survival. OS, overall survival. NA, not available.

## 5 Conclusion

Our findings demonstrate that an elevated serum IL-10/IL-6 ratio (≥2.30) at initial diagnosis serves as an independent predictor of SCNSL development and disease progression in DLBCL. Incorporating this ratio significantly enhances the prognostic accuracy of both CNS-IPI and NCCN-IPI scoring systems. These results underscore the potential of integrating inflammatory cytokine biomarkers with established clinical indices to refine risk stratification. Nonetheless, clinical translation of these biomarkers will require standardized cutoff definitions, multicenter validation, and longitudinal studies to monitor dynamic changes throughout disease progression and treatment. Further research is essential to address these gaps and realize their full clinical utility.

## Data Availability

The original contributions presented in the study are included in the article/**Supplementary Material**. Further inquiries can be directed to the corresponding author.
