# Supplementary material for: Elevated serum IL-10/IL-6 ratio as a novel biomarker for secondary central nervous system lymphoma and poor prognosis in DLBCL
Source: Front Immunol. 2025 Aug 13;16:1656044. doi: 10.3389/fimmu.2025.1656044 (PMC12380870; doi:10.3389/fimmu.2025.1656044)
Supplement: Supplementary file 1 [file DataSheet1.docx]

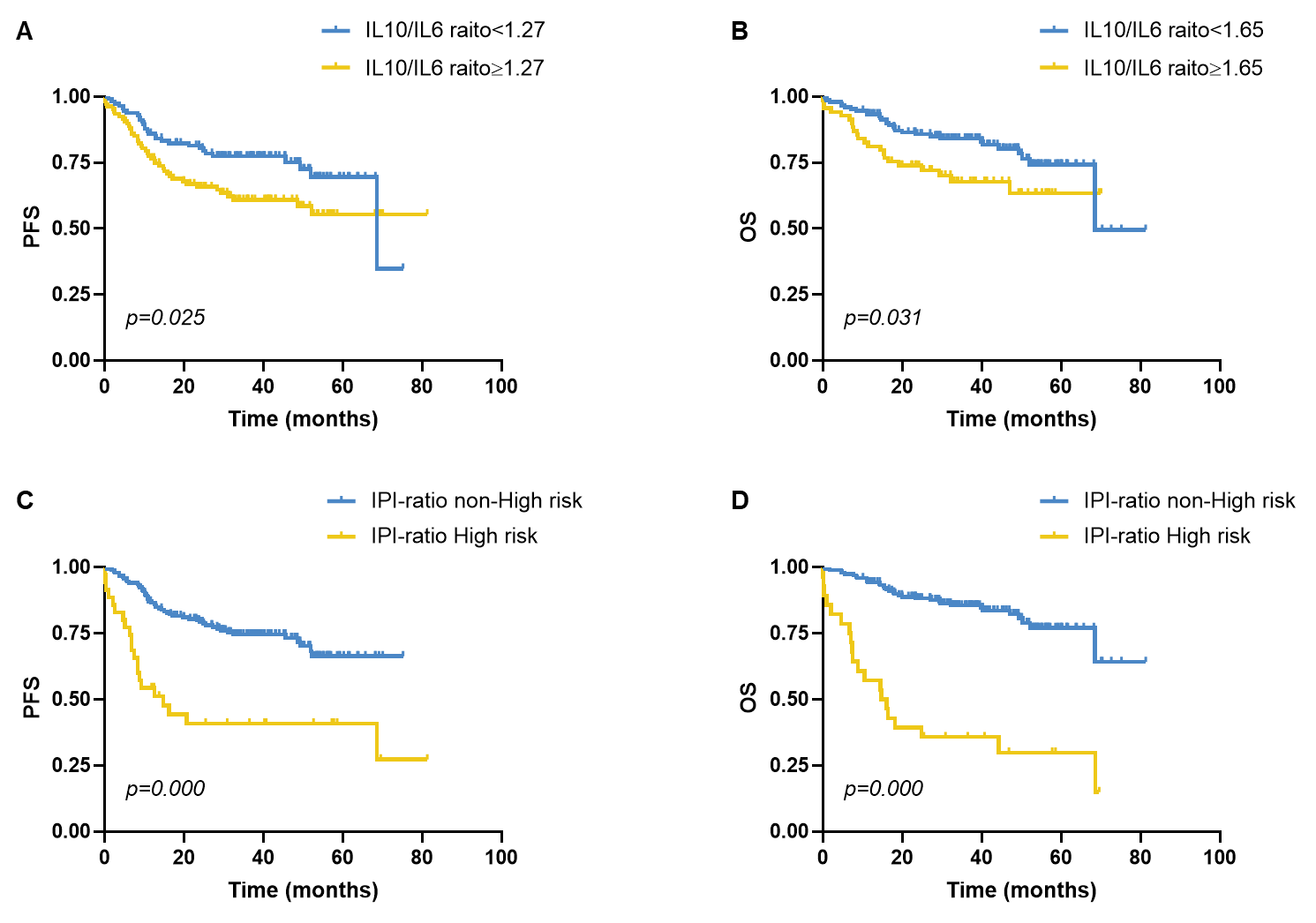


**Supplemental Figure 1.** Prognostic value of IL-10/IL-6 mRNA expression ratios in the LYSA cohort (GSE87371)

**(A-B)** Kaplan-Meier curves comparing **(A)** progression-free survival (PFS) and **(B)** overall survival (OS) between patients with high versus low IL-10/IL-6 mRNA expression ratios. Optimal cutoffs (PFS: 1.27; OS: 1.65) were determined by ROC analysis using disease progression and mortality as endpoints, respectively. **(C-D)** Integration of IL-10/IL-6 ratios (≥cutoff = 1 point) with IPI scores: The IPI-Ratio high-risk group (≥5 points) showed significantly worse **(C)** PFS and **(D)** OS compared to non-high-risk patients (<5 points). All survival analyses were performed using the Kaplan-Meier method with log-rank testing. *p* < 0.05 was considered statistically significant.
